# Variation of *Cicer* Germplasm to Manganese Toxicity Tolerance

**DOI:** 10.3389/fpls.2020.588065

**Published:** 2020-11-30

**Authors:** Karthika Pradeep, Richard W. Bell, Wendy Vance

**Affiliations:** Land Management Group, Agriculture Discipline, College of Science, Health, Engineering and Education, Murdoch University, Murdoch, WA, Australia

**Keywords:** chickpea (*Cicer arietinum* L.), genotypic variation, manganese toxicity scoring, manganese tolerance, solution culture, wild *Cicer*

## Abstract

After aluminum, manganese toxicity is the most limiting factor for crops grown in acidic soils worldwide. But overall, research on Mn toxicity is still limited. The poor acid tolerance of chickpea may be related to Mn toxicity, but there has been no previous screening of chickpea germplasm (nor in its wild *Cicer* relatives, *Cicer reticulatum* and *Cicer echinospermum*) for tolerance to Mn toxicity. A screening technique was developed for tolerance to Mn toxicity using three released cultivars of chickpea (*Cicer arietinum* L), Ambar, PBA HatTrick, and PBA Striker; one accession each of *C. reticulatum* and *C. echinospermum*; and lupin (*Lupinus angustifolius*) as a Mn-tolerant check, with eight Mn concentrations of 2, 25, 50, 100, 150, 200, 250, and 500 μM Mn as MnSO_4_ in a low-ionic-strength nutrient solution. The plants were harvested at 14 and 28 days after Mn treatments. The nutrient uptake in shoots (young, old leaves, and the rest of the shoot) and roots was investigated. The best discrimination between tolerant and intolerant *Cicer* genotypes based on relative shoot dry weight, root dry weight, total root length, and scoring of toxicity symptoms was achieved at 150 μM Mn after 14 days of growth in Mn solution. Among the chickpea cultivars, the greater relative plant growth (both shoot and root) of Ambar and PBA Striker at 100–200 μM Mn contrasted with that of PBA HatTrick, while the *C. echinospermum* accession was more tolerant to Mn toxicity than *C. reticulatum*. Manganese tolerance in both domestic cultivars and wild accessions was associated with internal tolerance to excess Mn following greater uptake of Mn and translocation of Mn from roots to shoots.

## Introduction

Manganese (Mn) toxicity is one of the important constraints limiting crop growth in acid soils worldwide. Manganese toxicity occurs because of increased Mn^2+^ concentration with increased acidity even through increased H^+^ in the root zone decreases the rate of Mn uptake by roots (Edwards and Asher, 1982). While aluminum (Al) toxicity is most common in severely acid soils, Mn is a likely growth-limiting factor in moderately–strongly acid soils, and the problem further worsens under waterlogged conditions and with environmental factors such as high soil temperature, which increases the concentration of Mn^2+^ (Foy et al., 1988; Khabaz-Saberi et al., 2010). Manganese toxicity generally occurs only at pH levels of 4.7 (CaCl_2_)/5.5 (H_2_O suspension) or below in well-drained soils, but in flooded and compacted soils, Mn can be toxic at a pH of 6 and higher if the soil parent material contains sufficient total Mn (Foy et al., 1988; Fernando and Lynch, 2015).

High concentrations of Mn in plant tissues alter enzymatic activity as well as uptake, redistribution, and use of other nutrients like Ca, Fe, Mg, N, and P (Santos et al., 2017; Li et al., 2019). Manganese toxicity reduced leaf CO_2_ assimilation rate, stomatal conductance, and leaf pigments (chlorophylls a and b) in different plant species in various studies (Stoyanova et al., 2009; Santos et al., 2017). Excess Mn enhanced production of reactive oxygen species (ROS) (Santos et al., 2017), triggering oxidative stress in plant cells (Demirevska-Kepova et al., 2004), and was associated with elevated peroxidase activity and lower activities of catalase, ascorbic acid oxidase, glutathione oxidase, and cytochrome C oxidase (Hannam and Ohki, 1988).

Differential Mn tolerances among plant genotypes within a species have been reported for soybean (*Glycine max*), wheat (*Triticum aestivum*), subclover (*Trifolium subterraneum*), bean (*Phaseolus vulgaris*), rice (*Oryza sativa*), cotton (*Gossypium hirsutum*), and cowpea (*Vigna unguiculata*) (Foy et al., 1988). The regulation of Mn uptake, translocation, and distribution are the main mechanisms of Mn toxicity tolerance. Transporters responsible for Mn acquisition and translocation and genes responsible for Mn detoxification have been identified in plant species such as *Arabidopsis* and rice (Tsunemitsu et al., 2018; Li et al., 2019). The ability of plant species to deal with high Mn may be by (1) limiting Mn absorption by the roots, (2) retaining Mn in the roots, or (3) tolerating high Mn in the shoots (Edwards and Asher, 1982; El-Jaoual and Cox, 1998; Blamey et al., 2015). In general, Mn-tolerant germplasms have tolerance to high internal tissue Mn, but in some species, low rates of Mn uptake and Mn retention in the root were important (Foy et al., 1988). For example, sunflower (*Helianthus annuus*) had high Mn uptake and internal tolerance but low root retention, whereas cassava (*Manihot esculenta*) had high root retention of Mn. In cowpea, Mn tolerance was associated with rapid uptake and translocation to tops (Foy et al., 1988).

Chickpea (*Cicer arietinum* L.) is an important pulse crop with an annual production of 11.5 million tonnes worldwide (Maesen et al., 2007; Merga and Haji, 2019). However, the yield of chickpea tends to be low and unstable, with a world average yield of 850 kg/ha (Merga and Haji, 2019), well below the estimated yield potential of 4,000 kg/ha (Singh et al., 1993; Singh and Ocampo, 1997). Part of the yield gap may be due to soil acidity constraints since the current pH (CaCl_2_) recommendation for chickpea cultivation is >5.5, and a surface pH of 5.0 is suitable only if subsurface pH is higher than 5.5 (Harries et al., 2005). Domestic chickpea cultivars are limited in their diversity, and traditional breeding methods have not produced cultivars with a large impact on chickpea production (Singh and Ocampo, 1997). Hence, wild *Cicer* can be exploited to develop resistant cultivars against stressors including low pH (Singh and Ocampo, 1997; Berger et al., 2003). Identifying acid tolerance in existing chickpea cultivars or in wild *Cicer* accessions, to develop more acid-tolerant cultivars, could increase the productivity and expand chickpea cultivation worldwide on acid soils.

Overall, screening for tolerance to acidic soils has focused on Al toxicity (Hayes et al., 2012). However, screening only for Al toxicity would be incomplete for improving chickpea tolerance to acid soils without ensuring that the germplasm also expressed tolerance to Mn toxicity. Interspecific and intraspecific differences in tolerance to Mn toxicity have been previously identified among crop plants providing potential to develop cultivars adapted to Mn stress in acid soils (Foy et al., 1988; Moroni et al., 2003). Chickpeas are considered very sensitive to Mn toxicity (GRDC, 2016), but research on response of chickpeas to Mn toxicity is very limited. Moreover, there are no previous screening studies reported on chickpea germplasm for Mn toxicity tolerance. The aim of this research was to develop a protocol for screening chickpeas in solution culture for Mn toxicity, so that wild *Cicer* accessions and chickpea germplasm can be ranked for Mn toxicity tolerance. Our main objectives were to (a) to determine the critical solution Mn level and duration of the experiment that differentiates tolerant and sensitive *Cicer* germplasm, (b) to identify suitable plant parameters that can identify Mn toxicity and can easily be used to rank a wide range of *Cicer* germplasm, and (c) to investigate how excess Mn^2+^ interacts with Ca^2+^ and Fe^2+^ in *Cicer* and the likely mechanism of tolerance to Mn excess.

## Materials and Methods

### Plant Material and Growth Conditions

A solution culture experiment was conducted in a growth cabinet set at 22°C and 12-h-day length. The light intensity at canopy height during the experiment as photosynthetically active radiation was around 500 μmol m^–2^ s^–1^. A split-plot experiment was designed with Mn treatments as main plots and cultivars as subplots. The experiment had five chickpea genotypes, including three released chickpea cultivars (Ambar, PBA HatTrick, and PBA Striker) and two wild *Cicer* species [*Cicer reticulatum* (AGG accession number 49936^1^) and *Cicer echinospermum* (accession number 50166^2^)]. *C. reticulatum* and *C. echinospermum* will be referred to as *C.retic* and *C.echino* throughout the manuscript. The rationale for inclusion of wild *Cicer* species in screening for abiotic stress tolerance traits and their collection environment were previously published (von Wettberg et al., 2018). Lupin (*Lupinus angustifolius*; variety Mandelup lupin) was included as a Mn-tolerant check (Blamey et al., 2015, 2017).

### Solution Composition

The ionic concentration of the solution, activity of Mn^2+^, and free activity of the ions were computed using a chemical speciation software, Geochem-EZ (Shaff et al., 2010). The basal nutrient solution used for this study had the following concentrations: Ca(NO_3_)_2_⋅4H_2_O, 400 μM; K_2_SO_4_, 350 μM; MgSO_4_⋅7H_2_O, 200 μM; NH_4_NO_3_, 100 μM; NH_4_H_2_PO_4_, 60 μM; H_3_BO_3_ 10 μM; ZnSO_4_⋅7H_2_O, 0.6 μM; CuSO_4_⋅H_2_O, 0.2 μM; and Na_2_MoO_4_⋅2H_2_O, 0.1 μM. Iron was added as 20 μM Fe-EDTA prepared from equimolar amounts of FeCl_3_⋅6H_2_O and Na_2_EDTA. The experiment had eight Mn treatments (in μM Mn): 2 (as control), 25, 50, 100, 150, 200, 250, and 500. Manganese was added as MnSO_4_⋅H_2_O. The pH of the initial nutrient solution was adjusted to 5.2 and maintained at 5.2 throughout the experiment with daily adjustments of 0.5 M HCl or NaOH. The experiment container had a capacity of 30 L, and the solution was renewed weekly.

### Seed Preparation and Experimental Setup

The seeds were initially scarified and then sterilized in 3% sodium hypochlorite for 5 min and thoroughly rinsed with deionized (DI) water. Seeds were germinated in wet paper towel in sealed plastic containers. Tap water was used to moisten the paper towel, and the containers were kept in dark conditions for 4 days at 22°C. The seeds were checked on alternate days for moisture and supplemented with water if needed. The germinated uniform seedlings were transferred into the lids of the experimental containers. The lids were made of 15-mm rigid white PVC foam, and strips of polyurethane foam held the seedlings into the lids of the experimental container. Six seedlings were transplanted for a genotype for a treatment, and the experiment was replicated thrice. The nutrient solution in the containers was aerated continuously. The plants were grown initially for 12 days in basal nutrient solution with 2 μM Mn (control) before adding Mn treatments. There were two harvests, 14 and 28 days after Mn treatment additions, and three plants per genotype at each harvest.

### Plant and Solution Measurements

The plants were scored for Mn toxicity symptoms weekly (at 7, 14, and 22 days) after Mn treatment additions to study the Mn effect over the experiment period, using a score card developed for chickpeas ranging from 0 (no symptoms) to 5 (very severe). Chlorophyll fluorescence measurements, Fv/Fm (variable fluorescence/maximum fluorescence) values, and the performance index (PI) were recorded for chickpea plants 3 weeks after Mn treatment additions using a Hansatech Handy PEA chlorophyll fluorometer. The measurements were made using leaf clips of 4-mm diameter on fully emerged leaves that had been dark adapted for 8 min. Dark adaptation was done using lightweight leaf clips which cover a section of the leaf.

Three plants per genotype were harvested at 14 and 28 days after Mn treatments and divided into shoot and root samples. The shoot was partitioned into old leaves (bottom two to three leaves from each branch), young leaves (top two to three leaves), and the rest of the shoot, and the remnant seeds were removed when weighing the samples. Roots from one of the plants were rinsed in DI water and stored in a refrigerator (4°C) before total root length, root volume, surface area, and root diameter measurements using the WinRHIZO root scanner. The shoot and root samples were dried in an oven for 3 days at 60°C to record shoot and root dry weight. Only the plant samples from Mn treatments of 2 μM (as control) and 100 to 200 μM (as they showed greater discrimination among genotypes for toxicity symptoms and plant growth) were acid digested using 70% conc. HNO_3_ in the digestion block for inductively coupled plasma (ICP) spectrometer analysis of Mn, Fe, and Ca. The following plant growth indices were calculated: relative shoot weight percentage, relative root weight percentage, relative root length, and root/shoot ratios.

Solution samples were collected during initial solution preparation and weekly at each renewal. The samples were filtered (using 0.45-μm filters) and acidified for ICP analysis of nutrients.

### Statistical Analysis

Statistics was performed using the GenStat Version 18 (VSN International, United Kingdom) analytical software. Analysis of variance (ANOVA) was computed using a split-plot model to assess the effect of Mn treatments and its interaction with genotypes. Manganese treatments were the main-plot factor, and genotypes were the subplot factor. Lupin data were included in computing the ANOVA. The data were log-transformed for parameters if they were not normally distributed. In such cases, log-transformed means along with back-transformed means in brackets were presented. The least significant difference, LSD (at *P* ≤ 0.05), was used to test for differences among the means of Mn treatments, genotypes, and their interactions. The level of significance (*P* values) and LSD_0__.__05_ values within and between Mn treatments were included only when the interactions (Mn treatments × genotypes) were significant.

## Results

### Nutrient Composition of the Solution

The nutrient solutions used in 15 previous Mn screening studies for various plant species were reviewed (data presented as Supplementary File, Table A). Solutions used in Mn screening studies varied in composition of basal nutrients, Mn concentrations, and pH levels, even among studies with the same plant species. For example, the solution used by Rout et al. (2001) to study Mn toxicity in rice was fourfold more concentrated than the nutrient solutions used by Shrestha et al. (2018). In order to mimic soil solution composition, the concentrations of macronutrients used in the current experiment were diluted when compared to other Mn screening studies in rice (Shrestha et al., 2018), triticale (Quartin et al., 1998), wheat (Khabaz-Saberi et al., 2010), and cotton (Foy et al., 1995) to name a few, and the pH of 5.2 was the average of 15 other Mn screening studies reviewed.

According to Geochem-EZ analysis, the ionic strength of nutrient solutions increased with Mn treatments from 3,287 μM (basal + 2 μM Mn) to 5,000 μM (basal + 500 μM Mn) (Table 1). About 90–94% of added Mn was present as free Mn^2+^ depending on Mn treatments. At solution renewal (weekly), Mn concentrations analyzed in the solution ranged between 70 and 92% of the nominated Mn concentrations in treatments of 50–500 μM Mn.

**TABLE 1:** Mn concentrations analyzed in nutrient solutions compared with Geochem-EZ Mn concentrations and activity and ionic strength.

**Added Mn concentrations (μM)**	**Actual Mn solution (μM) at start of ICP analysis**	**Actual Mn solution (μM) at 7 days after ICP analysis**	**Geochem-EZ-predicted Mn solution (μM)**	**Geochem-EZ-predicted% as free metal**	**Geochem-EZ-predicted free Mn activity (μM)**	**Geochem-EZ-predicted ionic strength of nutrient solution (μM)**
2	2.01	–	1.88	94.2	1.46	3,287
25	24.8	–	23.5	93.9	18.2	3,368
50	49.5	35.5	48.5	93.7	36.2	3,455
100	98.9	82.1	93.4	93.4	71.8	3,633
150	147	119	139	92.9	107	3,806
200	194	162	185	92.7	141	3,982
250	247	217	231	92.2	174	4,153
500	483	457	453	90.6	334	5,001

### Scoring of Plants for Mn Toxicity Symptoms

**FIGURE 1:**
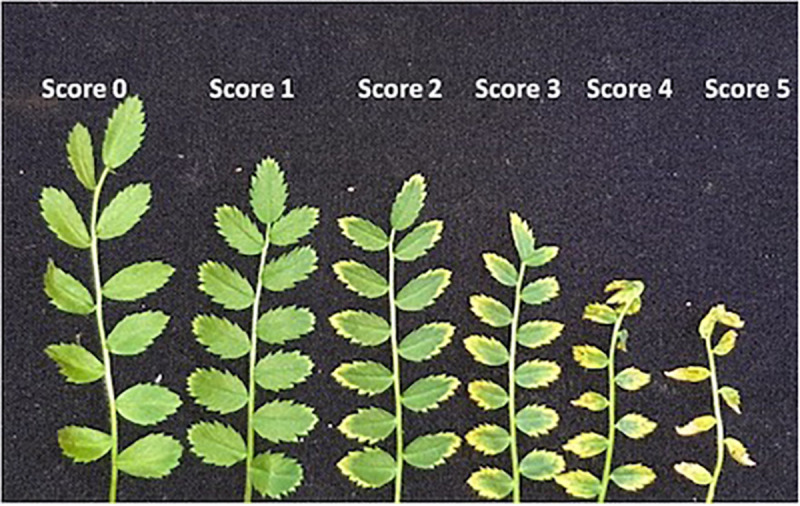
Scoring of *Cicer* sp. for Mn toxicity symptoms.

To rate Mn toxicity symptoms in *Cicer*, the plants were scored on a 0 to 5 scale (0: no symptoms, 1: very mild, 2: mild, 3: moderate, 4: severe, and 5: very severe). The detailed scoring system and pictorial descriptions are presented in the Supplementary File, Table B. In general, there was chlorosis in young leaves, and as the Mn toxicity increased, the chlorotic areas extended inwards toward the midrib, and there were brown necrotic spots in older leaves. With Mn > 150 μM, older leaves turned brown and had rusty-colored spots, and necrotic areas enlarged and covered most of the *Cicer* leaves except for an area around the base and midrib of leaflets. At high Mn toxicity levels, the young leaves were shriveled and curled, their size was significantly reduced, and leaflets dropped from the plants. Figure 1 shows the leaf symptoms of *Cicer* plants for Mn toxicity and the relevant scores.

**FIGURE 2:**
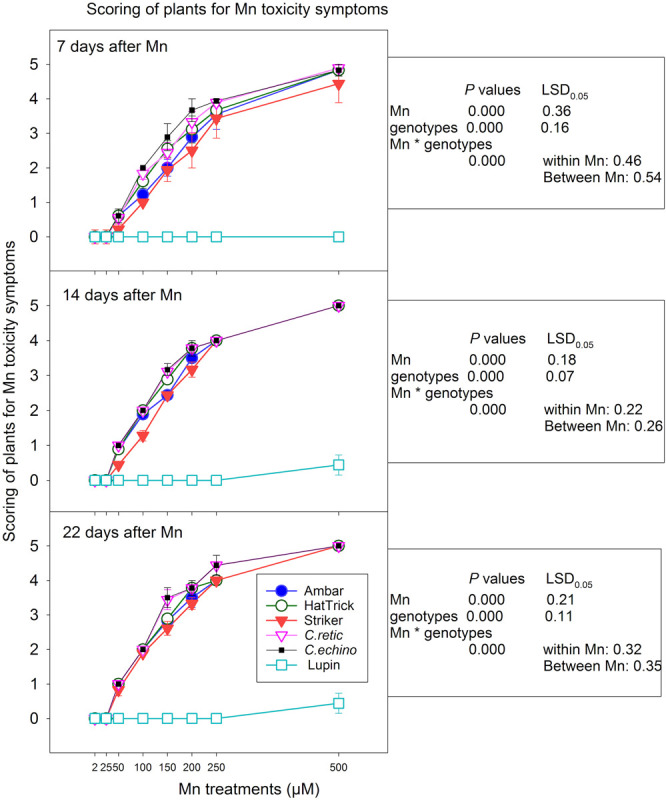
Scoring of *Cicer* genotypes and lupin for Mn toxicity symptoms at 7, 14, and 22 days after Mn additions. Vertical bars where visible represent the standard error of the mean. See Figure 1 for images of the symptoms for scores 0–5.

The *Cicer* plants grown in 2 and 25 μM Mn treatments showed no symptoms at 7, 14, and 22 days after Mn treatments (Figure 2). There was a significant increase in Mn toxicity scores with the increase in Mn levels from 50 to 500 μM (*P* < 0.0001) (Figure 2). The plants showed very mild symptoms with 50 μM Mn in all of the *Cicer* genotypes. With 100 μM Mn addition, there was differentiation among genotypes especially after 7 days of Mn addition, with PBA Striker scoring less compared to wild accessions. Similarly, with 150 μM Mn, the genotypes Ambar and PBA Striker showed mild Mn toxicity symptoms, whereas other genotypes showed moderate symptoms at 7 days after Mn treatment addition. The symptoms with 200 μM Mn were mostly moderate when scored at 7 days and increased to mostly severe symptoms at 22 days of Mn treatment. The symptoms on 7, 14, and 22 days after Mn were mostly severe and very severe at 250 and 500 μM Mn, respectively. The differentiation among genotypes was not obvious at 14 and 22 days after Mn scoring.

In general, mean scoring of genotypes across eight Mn treatments showed fewer symptoms in the PBA Striker, and the wild accessions showed more intense symptoms for Mn toxicity, and the severity increased with the increase in number of days of Mn exposure in all the genotypes. However, there was a significant correlation between scoring at 7 days and 14 days (*r* = 0.979) and 22 days (*r* = 0.972), as well as symptoms between 14 and 22 days (*r* = 0.993). Among the domestic cultivars, the PBA HatTrick showed more intense Mn toxicity symptoms compared with the PBA Striker. The scoring of Mn toxicity symptoms at 7 and 14 days after Mn treatments had a similar level of significant negative correlations with relative shoot dry weights (*r* = −0.92) and root dry weights (*r* = −0.84) at first harvest. Also, the scoring at 22 days was significantly correlated with relative shoot dry weight (*r* = −0.88) and root dry weight (*r* = −0.89) at the second harvest. Lupin showed no Mn toxicity symptoms at all Mn treatments except a very mild yellowing in two plants with 500 μM Mn treatment scored at 14 and 22 days after Mn.

### Plant Growth

#### Shoot Dry Weight

**FIGURE 3:**
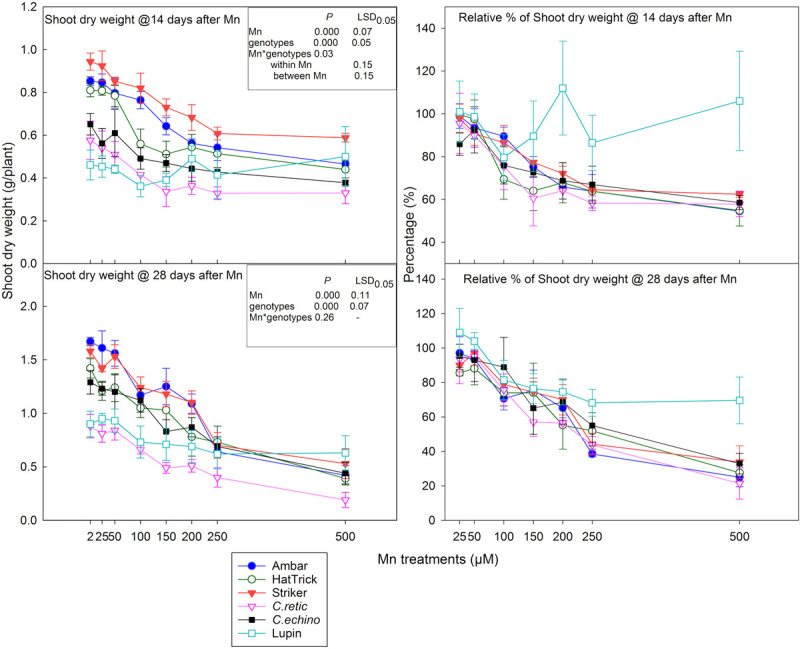
Shoot dry weight at 14 and 28 days after Mn treatments and their relative shoot dry weight percentage (in relation to 2 μM) of five *Cicer* genotypes and lupin. Vertical bars where visible represent the standard error of the mean.

At 14 days after Mn addition, there was a significant decrease in shoot dry weight (*P* < 0.001), and the interaction between treatment and genotypes was significant (*P* = 0.03). In general, among the *Cicer* genotypes, the PBA Striker had higher mean shoot dry weight while both wild *Cicer* accessions had the least shoot dry weight across all Mn treatment levels (Figure 3). The shoot dry weight at both harvests was not significantly different at 25 and 50 μM Mn compared to the control. The relative reduction in shoot dry weight in *Cicer* ranged between 21 and 42% with 100–500 μM Mn. The Mn concentrations, 100–200 μM Mn, showed better discrimination in relative shoot dry weight among the *Cicer* genotypes (Figure 3). At 100 μM Mn, the PBA HatTrick showed the highest relative reduction of 30%. However, at 150 μM Mn, *C.retic* showed greater shoot dry weight reduction (39%) compared to *C.echino* (27%); cultivars PBA HatTrick, Ambar, and PBA Striker showed 36, 25, and 23% reductions, respectively. The reduction in shoot dry weight at the highest Mn, 500 μM, was 38% or more in *Cicer* genotypes. In general, lupin did not show consistent change in shoot dry weight with Mn additions.

In general, shoot dry weight at 28 days after Mn treatment additions followed a similar trend as that at 14 days; however, the interaction between Mn treatments and genotypes was not significant (*P* = 0.127). The reduction in dry weight due to Mn toxicity intensified at the second harvest, especially at high Mn treatment levels (Figure 3). The mean relative reduction in shoot dry weight at 100 μM was 22%, and at 500 μM, it increased to 72%. Mean shoot dry weight measured across eight Mn treatments for the PBA HatTrick was significantly less than that for Ambar and PBA Striker. Lupin showed 18–30% relative reduction in shoot dry weight when Mn concentrations in the solution increased from 100 to 500 μM.

#### Root Dry Weight

**FIGURE 4:**
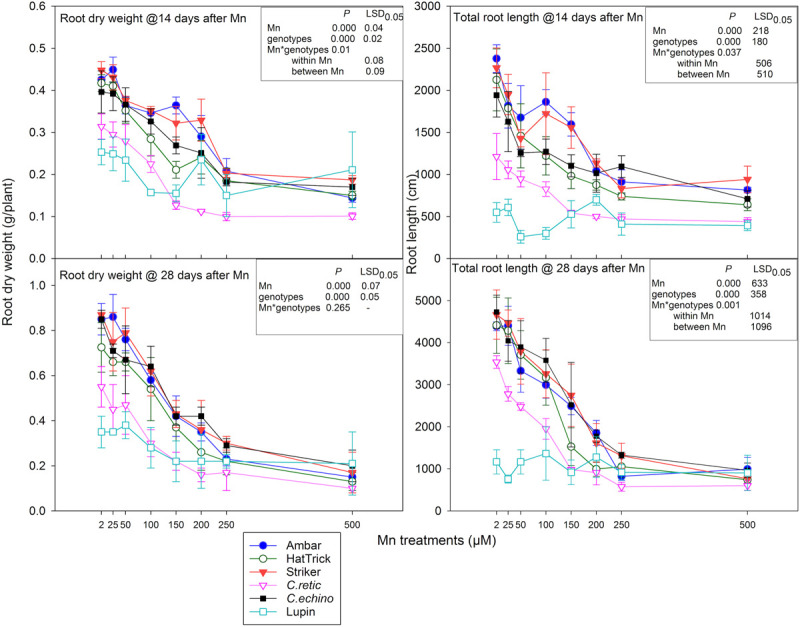
Root dry weight (g/plant) and total root length (cm/plant) at 14 and 28 days after Mn treatments of *Cicer* genotypes and lupin. Vertical bars where visible represent the standard error of the mean.

The interaction of root dry weight measured at 14 days after Mn additions was significant between Mn treatment and genotypes (*P* = 0.01), and the relative reduction in root dry weight due to Mn toxicity was greater than shoot dry weight in *Cicer* genotypes (Figures 3, 4). The mean reduction in root dry weights at 250 and 500 μM Mn was 20% higher than that in shoot dry weight. However, there was a strong positive correlation between shoot and root dry weight measured at 14 days after Mn treatments (*r* = 0.829). Relative shoot dry weight and root dry weight of wild *C.retic* accession and the domestic cultivar PBA HatTrick were less than that for other genotypes, especially at 150 μM and above. At 100 μM Mn, the reduction in root dry weight ranged between 13% in *C.echino* and 33% in PBA HatTrick, whereas at 200 μM Mn, PBA Striker showed comparatively low root weight reduction of 26% compared with other genotypes. The reduction in root dry weight was high in *C.retic*, ranging between 58 and 68%, in Mn concentrations of 150 μM and above. Among the domestic cultivars, PBA HatTrick was affected more by Mn toxicity than were Ambar and PBA Striker at levels of 100 and 200 μM with 10–20% more root dry weight reduction. However, at high Mn levels, 250 and 500 μM, there was less differentiation among the *Cicer* genotypes. The mean root dry weight of eight Mn levels for PBA HatTrick was significantly lower than that for other domestic cultivars, while wild accession *C.retic* had the lowest mean root weight among the *Cicer* genotypes. Lupin showed inconsistent responses to Mn additions (Figure 4).

At the second harvest, there was a 15% greater reduction in relative root dry weight of *Cicer* genotypes than at 14 days at 150 μM Mn and above. However, there was no significant interaction between Mn treatments and genotypes (*P* = 0.265). Similar to shoot dry weight, at 25 and 50 μM, the mean root dry weight was not significantly different from control at both harvests. With the increase in Mn concentrations from 100 to 500 μM, the mean reduction in root dry weight increased from 29 to 80% (Figure 4). Lupin showed around 50% root dry weight reduction at 500 μM Mn. There was a significant correlation between root dry weight at the first and second harvests (*r* = 0.82) and also between shoot and root dry weight at the second harvest (*r* = 0.88). At both harvests, *C.echino* had higher root/shoot ratio than other *Cicer* genotypes (data not presented).

#### Total Root Length

The total root length measured after 14 days of Mn treatments responded to the interaction between Mn and genotypes (*P* = 0.04). The mean root length across Mn treatments for genotypes Ambar and PBA Striker was significantly greater than that of other genotypes, and wild *C.retic* accession had the least root length. With Mn increase from 2 to 500 μM in solutions, the decline in total root length ranged between 10 and 64% in *Cicer* genotypes (Figure 4). At 100 μM Mn, PBA HatTrick showed higher reduction in total root length, and at 150 μM Mn, the decline was greater in PBA HatTrick (51%) and *C.retic* (54%) than in other genotypes. The relative reduction in root length was less in *C.echino* at 150–250 μM Mn than in *C.retic* (Figure 4).

Similar to root weight, the reduction in total root length was greater at the second harvest than at the first harvest, especially at 150 μM Mn and above (Figure 4). The interaction between Mn treatments and genotypes was significant (*P* = 0.000) for total root length at the second harvest. At 100 and 150 μM Mn, *C.retic* showed significant root length reduction compared with other *Cicer* genotypes; at 150 μM Mn, *C.retic* had 32, 28, and 23% greater relative root length reduction than genotypes PBA Striker, Ambar, and *C.echino*, respectively. At 250 and 500 μM Mn, the relative reduction in root length was more than 70 and 75%, respectively, in all the *Cicer* genotypes, while lupin showed only 25% reduction in root length at 500 μM Mn.

### Chlorophyll Fluorescence Measurements

**FIGURE 5:**
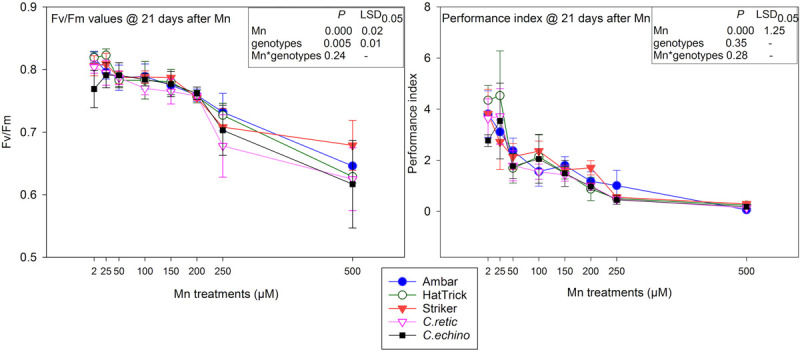
Fv/Fm values and performance index (PI) of five *Cicer* genotypes in eight Mn treatment levels measured at 21 days after Mn additions. Vertical bars where visible represent the standard error of the mean.

The mean variable fluorescence/maximum fluorescence (Fv/Fm) values of *Cicer* genotypes for 150 μM Mn and above were significantly lower than that of the control (Figure 5). However, the interaction between Mn treatments and *Cicer* genotypes for Fv/Fm measured at 3 weeks after Mn treatments was not significant (*P* = 0.242). The highest Mn treatment, 500 μM, reduced Fv/Fm measurements in *Cicer* genotypes by only 22%. The chlorophyll fluorescence measurements as Fv/Fm were negatively correlated with Mn toxicity scores at 7 days (*r* = −0.79), 14 days (*r* = −0.72), and 22 days (*r* = −0.72).

PI measurements were more sensitive to Mn toxicity than were Fv/Fm values in *Cicer* genotypes with a significant reduction in mean values of PI for Mn treatments 50 μM and above (Figure 5). There was 45% reduction in PI at 50 μM Mn and up to 94% at 500 μM Mn. However, there was no significant interaction between Mn treatments and genotypes (*P* = 0.2819). Similar to Fv/Fm, there were significant negative correlations between PI values and plant scores at 7 days after Mn (-0.73), 14 days (*r* = −0.75), and 22 days (*r* = −0.750).

### Ion Concentrations in Leaves and Roots

#### Mn Concentrations and Uptake

Manganese concentrations in young leaves increased with an increase in Mn solution concentrations (Table 2); among the genotypes, the Mn increase in wild *Cicer* accessions and PBA Striker was higher than that in Ambar and PBA HatTrick at both harvests when compared to the control. Among the genotypes, PBA Striker and *C.echino* had significantly higher mean Mn concentration in young leaves at the first harvest compared with other *Cicer* genotypes (*P* ≤ 0.05); however, there was no significant difference in young leaf Mn concentrations among the *Cicer* genotypes at the second harvest.

**TABLE 2:** Log-transformed Mn concentrations in young and old leaves of *Cicer* genotypes and lupin grown in 2, 100, 150, and 200 μM Mn at 14 and 28 days after Mn treatments [back-transformed means (mg/kg) presented in brackets].

**Log_10_ transformed Mn concentrations at 14 days after Mn treatment**								
	**Young leaves**				**Old leaves**			
**Mn levels (μM)**	**2**	**100**	**150**	**200**	**2**	**100**	**150**	**200**

Ambar	2.18 (153)	3.31 (2,080)	3.39 (2,450)	3.51 (3,238)	2.33 (214)	3.61 (4,092)	3.71 (5,081)	3.75 (5,597)
HatTrick	2.17 (151)	3.33 (2,147)	3.43 (2,675)	3.53 (3,413)	2.36 (230)	3.58 (3,793)	3.53 (3,396)	3.68 (4,875)
Striker	2.20 (160)	3.38 (2,427)	3.50 (3,152)	3.38 (4,278)	2.32 (210)	3.60 (3,962)	3.62 (4,197)	3.73 (5,432)
*C.retic*	2.06 (117)	3.31 (2,064)	3.41 (2,577)	3.41 (3,331)	2.20 (159)	3.33 (2,158)	3.53 (3,396)	3.38 (2,415)
*C.echino*	2.07 (117)	3.36 (2,282)	3.36 (3,262)	3.51 (4,192)	2.25 (179)	3.56 (3,614)	3.76 (5,780)	3.75 (5,636)
Lupin	2.61 (412)	3.69 (4,991)	3.83 (6,850)	3.84 (8,061)	2.69 (490)	3.81 (6,441)	3.65 (4,487)	4.02 (1,0471)

	***P* values**		**LSD_0__.05_**		***P* values**		**LSD_0__.05_**	

Mn treatment	0.000		0.13		0.000		–	
Genotypes	0.000		0.05		0.000		–	
Mn * genotypes	0.21		–		0.008		within Mn: 0.07, between Mn: 0.07	

**Log_10_ transformed Mn concentrations at 28 days after Mn treatment**								

	**Young leaves**				**Old leaves**			
**Mn levels (μM)**	**2**	**100**	**150**	**200**	**2**	**100**	**150**	**200**

Ambar	2.27 (187)	3.40 (2,498)	3.44 (2,753)	3.47 (2,982)	2.61 (413)	3.62 (4,217)	3.76 (5,768)	3.81 (6,531)
HatTrick	2.26 (183)	3.28 (1,922)	3.39 (2,460)	3.59 (3,907)	2.51 (326)	3.56 (3,664)	2.51 (4,064)	3.81 (6,427)
Striker	2.22 (165)	3.30 (1,985)	3.43 (2,721)	3.59 (3,881)	2.46 (288)	3.58 (3,793)	2.46 (5,081)	3.74 (5,495)
*C.retic*	2.16 (145)	3.31 (2,059)	3.45 (2,861)	3.53 (3,442)	2.42 (264)	3.31 (2,070)	2.42 (2,454)	3.55 (3,540)
*C.echino*	2.13 (136)	3.31 (2,036)	3.48 (3,049)	3.53 (3,417)	2.34 (221)	3.58 (3,801)	2.34 (3,802)	3.86 (7,328)
Lupin	2.72 (529)	3.66 (4,641)	3.80 (6,394)	3.95 (8,908)	2.82 (654)	3.84 (6,998)	4.03 (6,998)	4.19 (15,523)

	***P* values**		**LSD_0__.05_**		***P* values**		**LSD_0__.05_**	

Mn treatment	0.000		0.18		0.000		0.13	
Genotypes	0.000		0.06		0.000		0.08	
Mn * genotypes	0.08		–		0.27		–	

The old leaves had higher Mn concentrations than had the young leaves, and Mn concentrations increased significantly with an increase in Mn treatment levels (*P* ≤ 0.05; Table 2). Among the *Cicer* genotypes, *C.retic* had significantly lower mean Mn concentration in the old leaves at both harvests compared with the others. The mean increase in Mn concentrations with treatments of 100–200 μM Mn when compared to the control was lower in PBA HatTrick and *C.retic* than in others. The old/young leaves ratios of Mn concentrations were lower in *C.retic* at both harvests (data not shown). Lupin had significantly higher mean Mn concentrations in leaf tissues compared with *Cicer* genotypes.

Manganese concentrations in roots increased with an increase in Mn treatment levels (*P* ≤ 0.05), and there was a significant cultivar effect. In contrast to Mn concentrations in shoot and leaf tissues, *C.echino* accession had a lower mean root Mn concentration than *C.retic* at both harvests, and PBA Striker had lower Mn concentrations in roots than Ambar (Table 3).

**TABLE 3:** Manganese concentrations in roots (mg/kg) and log-transformed shoot/root Mn ratios of *Cicer* genotypes and lupin grown in 2, 100, 150, and 200 μM Mn at 14 and 28 days after Mn treatments (back-transformed means for shoot/root ratios presented in brackets).

**Root Mn concentrations (mg/kg) and shoot/root Mn ratios at 14 days after Mn treatment**								
	**Root**				**Log_10_ shoot/root Mn**			
**Mn levels (μM)**	**2**	**100**	**150**	**200**	**2**	**100**	**150**	**200**

Ambar	586	4,366	4,700	6,466	−0.14 (0.72)	0.14 (1.40)	0.11 (1.29)	0.08 (1.22)
HatTrick	390	3,966	3,600	6,966	−0.08 (0.81)	0.10 (1.27)	0.25 (1.78)	0.08 (1.21)
Striker	176	3,800	3,900	6,233	0.28 (1.89)	0.22 (1.66)	0.25 (1.78)	0.23 (1.70)
*C.retic*	253	3,866	4,233	7,433	0.01 (1.02)	−0.02 (0.95)	0.20 (1.61)	0.07 (1.17)
*C.echino*	135	1,766	2,900	4,433	0.26 (1.82)	0.32 (2.11)	0.40 (2.51)	0.28 (1.90)
Lupin	403	3,633	4,100	5,900	0.29 (1.94)	0.44 (2.75)	0.34 (2.17)	0.46 (2.93)

	***P* values**		**LSD_0__.05_**		***P* values**		**LSD_0__.05_**	

Mn treatment	0.007		2,537		0.40		–	
Genotypes	0.000		728		0.000		0.14	
Mn * genotypes	0.33		–		0.81		–	

**Root Mn concentrations (mg/kg) and shoot/root Mn ratios at 28 days after Mn treatment**								

	**Root**				**Log_10_ shoot/root Mn**			
**Mn levels (μM)**	**2**	**100**	**150**	**200**	**2**	**100**	**150**	**200**

Ambar	480	1,833	4,100	4,100	0.04 (1.10)	0.48 (3.04)	0.43 (2.70)	0.49 (3.13)
HatTrick	290	2,066	3,333	3,666	0.19 (1.55)	0.36 (2.32)	0.35 (2.32)	0.57 (3.69)
Striker	253	1,933	2,266	4,000	0.11 (1.30)	0.35 (2.25)	0.62 (4.19)	0.52 (3.28)
*C.retic*	346	2,866	3,833	6,100	−0.004 (0.99)	0.16 (1.45)	0.15 (1.40)	0.25 (1.77)
*C.echino*	185	1,600	2,766	3,600	0.20 (1.60)	0.44 (2.74)	0.39 (2.44)	0.45 (2.81)
Lupin	271	2,766	2,533	5,433	0.74 (5.50)	0.67 (4.72)	0.81 (4.72)	0.81 (6.56)

	***P* values**		**LSD_0__.05_**		***P* values**		**LSD_0__.05_**	

Mn treatment	0.001		1,370		0.01		0.14	
Genotypes	0.04		823		0.000		0.19	
Mn * genotypes	0.45		–		0.93		–	

The mean shoot Mn content of *C.retic* was significantly lower than that of other genotypes; however, the root Mn uptake of *C.retic* was not different from others. At 100–200 μM, the relative increase in Mn uptake (both shoot and root) due to Mn treatments was higher in PBA Striker and *C.echino* than in others (Supplementary File, Table C). The mean shoot/root Mn ratios at 14 days after Mn treatments of genotypes PBA Striker and *C.echino* accession were higher than those of other *Cicer* genotypes. However, at 28 days, shoot/root Mn ratios of *C.retic* was lower than that of other genotypes (Table 3).

#### Ca Concentrations and Uptake

Calcium concentrations in young leaves at both harvests decreased with the increase in Mn solution concentrations (*P* ≤ 0.05); however, the interaction between treatment and cultivar was not significant (Table 4). Among the *Cicer* genotypes, *C.echino* accession and PBA HatTrick had high and low mean Ca concentrations in young leaves, respectively. The mean percentage reduction of Ca concentrations across the five *Cicer* genotypes measured in young leaves at the first harvest in Mn treatments 100, 150, and 200 μM Mn were 29, 38, and 40%, respectively, when compared to control. At the second harvest, the mean reduction in Ca concentrations was 8% more than that at the first harvest.

**TABLE 4:** Ca concentrations (mg/kg) in young leaves and roots of *Cicer* genotypes and lupin grown in solutions at 2, 100, 150, and 200 μM Mn for 14 and 28 days after Mn treatments.

**Ca concentrations (mg/kg) at 14 days after Mn treatment**									
	**Young leaves**					**Root**			
**Mn levels (μM)**	**2**	**100**		**150**	**200**	**2**	**100**	**150**	**200**

Ambar	9,533	5,933		5,000	4,767	4,466	4,366	4,433	4,066
HatTrick	7,600	5,866		5,166	4,700	4,100	4,266	4,466	3,900
Striker	9,266	7,067		5,833	6,067	5,000	4,133	4,166	4,233
*C.retic*	9,433	6,433		5,266	5,000	4,133	3,433	3,666	3,566
*C.echino*	9433	6,833		6,400	6,533	4,233	3,533	3,833	3,466
Lupin	18,666	14,000		12,500	11,000	4,533	4,666	4,600	4,133

	***P* values**	**LSD_0__.05_**				***P* values**	**LSD_0__.05_**		

Mn treatment	0.004	1,829				0.26	–		
Genotypes	0.000	840				0.000	351		
Mn * genotypes	0.20	–				0.64	–		

**Ca concentrations (mg/kg) at 28 days after Mn treatment**									

	**Young leaves**					**Root**			
**Mn levels (μM)**	**2**	**100**		**150**	**200**	**2**	**100**	**150**	**200**

Ambar	10,300	6,866		5,533	3,866	4,500	4,233	4,800	4,100
HatTrick	8,467	5,233		4,366	4,833	3,966	4,200	4,500	4,100
Striker	8,950	6,000		4,933	5,400	4,500	4,100	4,733	4,100
*C.retic*	9,800	5,733		5,466	4,866	4,433	4,500	4,000	3,800
*C.echino*	9,767	6,000		6,100	4,600	4,533	4,100	3,933	3,500
Lupin	23,333	13,500		13,000	12,500	4,766	3,966	2,783	3,933

	***P* values**		**LSD_0__.05_**			***P* values**	**LSD_0__.05_**		

Mn treatment	0.0001		1,281			0.21	–		
Genotypes	0.000		800			0.19	–		
Mn * genotypes	0.000		within Mn: 1,601, between Mn: 1,815			0.16	–		

The Ca concentration in root tissues of *Cicer* genotypes at both harvests was not affected by the increase in Mn concentrations in solutions (*P* ≥ 0.05) (Table 4). Among the genotypes, the mean Ca concentrations in roots of wild *C.retic* and *C.echino* accessions were lower than those in chickpea cultivars at the first harvest; the mean root Ca concentrations in *C.retic* and *C.echino* were 18 and 16% less than that in PBA Striker, respectively.

The shoot Ca uptake declined significantly with increasing Mn solution in *Cicer* genotypes (*P* ≤ 0.05); the wild *C.retic* accession showed a significant mean reduction of shoot Ca (63%), and its mean shoot Ca content was significantly lower than that of other *Cicer* genotypes. Chickpea cultivars PBA Striker and Ambar had high mean shoot Ca uptake compared with the PBA HatTrick (data presented as Supplementary File, Table C). Similarly, the root Ca uptake decreased with Mn treatments; however, the percentage decrease was smaller than that in shoots.

Mn/Ca ratios in shoot and root increased with an increase in Mn levels in treatment solutions, and wild *C.retic* accession had lower and higher mean Mn/Ca ratios in shoot and root, respectively, compared with other chickpeas. The results and data are presented in Appendix A as Supplementary Data (Table D).

#### Fe Concentrations and Uptake

Manganese treatments had a significant interaction with genotypes for Fe concentrations in young leaves at the first harvest (*P* ≤ 0.05) (Table 5). Manganese treatments had a significant effect on shoot Fe uptake (*P* ≤ 0.05) with 25–58% reduction at Mn levels of 100 to 200 μM at both harvests. The mean reduction in shoot Fe uptake of *C.echino* accession and PBA Striker was very less compared to that in other genotypes (Supplementary File, Table C). The mean Fe concentrations in young leaves of *C.retic* and *C.echino* accessions were 15 and 19% and 21 and 35% higher than Ambar at the first and second harvests, respectively. In lupin, Fe concentrations in young leaves were twice as high as those in *Cicer* genotypes. Root Fe concentrations at the second harvest showed a significant increase due to Mn (*P* ≤ 0.05) (Table 5). In contrast to young leaves’ Fe concentrations, the *C.echino* accession had significantly lower Fe in roots than had Ambar at both harvests.

**TABLE 5:** Fe concentrations (mg/kg) in young leaves and roots of *Cicer* genotypes and lupin grown in solutions at 2, 100, 150, and 200 μM Mn for 14 and 28 days after Mn treatments.

**Fe concentrations (mg/kg) at 14 days after Mn treatment**								
	**Young leaves**				**Root**			
**Mn levels (μM)**	**2**	**100**	**150**	**200**	**2**	**100**	**150**	**200**

Ambar	146	104	80	84	2,400	3,200	3,400	3,833
HatTrick	143	99	102	89	1,900	2,666	3,233	3,700
Striker	116	108	107	88	2,066	2,400	2,900	2,400
*C.retic*	136	123	122	103	2,866	4,100	3,900	4,133
*C.echino*	129	143	145	95	1,833	2,300	2,466	2,500
Lupin	280	226	235	123	1,160	2,033	2,233	2,600

	***P* values**	**LSD_0__.05_**			***P* values**	**LSD_0__.05_**		

Mn treatment	0.13	–			0.21	–		
Genotypes	0.001	24			0.000	773		
Mn * genotypes	0.02	within Mn: 48, between Mn: 65			0.99	–		

**Fe concentrations (mg/kg) at 28 days after Mn treatment**								

	**Young leaves**				**Root**			
**Mn levels (μM)**	**2**	**100**	**150**	**200**	**2**	**100**	**150**	**200**

Ambar	151	112	97	102	2,300	4,633	4,866	4,800
HatTrick	134	133	100	120	2,000	3,366	3,466	3,266
Striker	140	115	106	102	2,100	3,233	3,600	3,400
*C.retic*	157	153	153	124	2,400	4,400	3,800	4,033
*C.echino*	128	220	190	180	1,866	3,600	3,533	2,966
Lupin	205	170	128	170	1,800	1,933	1,046	1,800

	*P* values	LSD_0__.05_			*P* values	LSD_0__.05_		

Mn treatment	0.90	–			0.01	769		
Genotypes	0.001	33			0.000	696		
Mn * genotypes	0.33	–			0.42	–		

There was a significant interaction between Mn treatments and cultivars (*P* ≤ 0.05) for shoot Mn/Fe ratio at both harvests: wild *C.retic* and *C.echino* accessions had lower Mn/Fe ratio in shoots compared to domestic cultivars (Supplementary Data).

## Discussion

The screening of *Cicer* plants for Mn toxicity was not previously reported, and depending on the plant criterion used, concentrations of 50–100 μM Mn were toxic. The median toxic concentration of Mn required to reduce plant growth was 46 μM from studies of a range of species (Kopittke et al., 2010). Chickpeas were more sensitive to Mn toxicity than other species like sunflower (Blamey et al., 2015), triticale (Quartin et al., 1998), oats (Clark, 1983), narrow-leaved and white lupin (Blamey et al., 2015), and rice (Rout et al., 2001) where a Mn concentrations of 100 μM had no effect on plant growth. On the other hand, chickpeas had a similar level of tolerance to rapeseed genotypes (*Brassica napus* and *Brassica rapa*) (Moroni et al., 2003) and was more tolerant to Mn toxicity than barley (Williams and Vlamis, 1957) and cowpea and soybean (Blamey et al., 2017), which makes chickpeas suitable for cultivation in moderately acidic soils with soil solution Mn levels <100 μM. To differentiate among *Cicer* genotypes (both domestic cultivars and wild species) in tolerance to Mn toxicity, 100–200 μM Mn was identified in this study.

High-ionic-strength solutions will alter the outcomes of Mn toxicity studies as the high concentrations of nutrients will decrease Mn availability due to formation of complexes with Mn (Kopittke et al., 2010). The ionic concentration of the solution used in the present study was relatively low (ranging between 3,000 and 5,000 μM), compared to most of the Mn toxicity screening studies that commonly used Hoagland solution (26,000 μM). Actual Mn concentrations in solutions analyzed at day 1 and solution renewal (day 7) of the treatment additions were very close to required Mn treatments in the present study. Furthermore, the Geochem-EZ analysis of nutrient solution confirmed more than 90% availability of Mn in solutions with minimal loss of Mn^2+^ due to formation of complexes with metals and ligands. Similarly, using Geochem-EZ, we calculated that in Mn screening studies by Andrew and Hegarty (1969) and Rout et al. (2001), both with around 23,000 μM ionic concentration (for 182 and 142 μM Mn treatments, respectively), 86% of Mn^2+^ was available as a free metal for plant uptake, and in Quartin et al. (1998), with an ionic concentration of 7,000 μM (200 μM Mn), 90% of Mn^2+^ was available as a free metal for plant uptake. The ionic strength of the solution in this study reflects the soil solution concentration of a highly weathered Oxisol from Queensland, which is around 5,000 μM in soil extracts (Kopittke et al., 2010). The solution pH of 5.2 was maintained for the present study, which was appropriate for assessing Mn toxicity, but not as acidic as Al toxicity studies where usually a pH of <4.5 is used (Kopittke et al., 2011).

Additions of Ca, Fe, Al, Si, and NH_4_^+^ to the growth medium have been shown to ameliorate Mn toxicity in plants (Foy et al., 1988). For instance, silicon addition decreased Mn toxicity symptoms in soybean and cucumber (*Cucumis sativus* L.) and improved plant growth by preventing localized accumulation of Mn in leaves (Shi et al., 2005; Liu et al., 2019), and an increase in Fe supply in the solution ameliorated Mn toxicity in sunflower and soybean by decreasing Mn uptake and translocation (Blamey et al., 2019). The nutrient solution used in this study had no Si, and other nutrients were at low concentrations, making Mn in this solution more toxic than if Si was added. In studies by Rout et al. (2001) and Quartin et al. (1998), 86 and 92% of Ca^2+^ from 4,000 and 1,000 μM in solutions were available for plants according to Geochem-EZ, that is, 3,600 and 600 μM more Ca^2+^, respectively, compared with the present study.

Manganese toxicity in plants generally occurs as leaf chlorosis and crinkling symptoms; however, genetics, environmental conditions (such as light exposure and temperature), and Mn levels affect the type and extent of the symptoms (Fernando and Lynch, 2015), and the necrotic margins may be due to accumulation of excess Mn oxides and phenols in the cell wall (Horiguchi, 1987). Even though the wild *C. echinospermum* accession showed better tolerance to Mn toxicity than did *C. reticulatum* in terms of relative plant growth, they scored the same for Mn toxicity symptoms. The wild accessions were the first to show symptoms of Mn toxicity. Cultivars PBA Striker and Ambar had better relative plant growth compared with PBA HatTrick, and they scored lower for Mn toxicity symptoms. Scoring of visual symptoms could be helpful in diagnosis of Mn toxicity differences among domesticated chickpea cultivars. However, scoring alone was not a satisfactory parameter to rank wild accessions for Mn tolerance as the extent of the symptom severity was not closely related to plant growth response in wild accessions. Moreover, the toxicity symptoms increased with the duration of plant exposure to Mn treatments in all the genotypes, making it necessary to score on a fixed day after treatment to be a reliable parameter for ranking genotypes.

Chlorophyll fluorescence measurements have been used to assess the function of photosystem II of photosynthesis under Mn toxicity since the structural and functional integrity of chloroplasts is damaged by excess Mn (Hajiboland and Hasani, 2007). However, in the present study, Fv/Fm ratios measured at 21 days after Mn addition declined only at 150 μM Mn and did not discriminate among genotypes as well as visual scoring of intensity of symptoms or plant biomass measurements. Similarly, periodical measurements of Fv/Fm ratios did not predict the appearance of Mn toxicity symptoms in olive (*Olea europaea*) leaves (Chatzistathis et al., 2011), and Fv/Fm ratios with Mn treatments of 50 and 100 μM did not show any inhibition in rice and sunflower (Hajiboland and Hasani, 2007). In this study, the inhibition in plant growth measurements due to excess Mn was not reflected in the chlorophyll fluorescence response of *Cicer*, which suggests that growth and dry matter production in response to Mn toxicity are not determined by direct inhibition of photosynthesis but rather by other factors like oxidative damage and metabolic disturbances (Li et al., 2019), requiring further investigation in *Cicer*.

The present methodology of growing seedlings for several days before starting Mn screening was followed in similar works with soybean (Santos et al., 2017), ryegrass (Inostroza-Blancheteau et al., 2017), wheat (Khabaz-Saberi et al., 2010), and rice (Dziwornu et al., 2018). Also, the duration of the experiment that exposed plants to Mn^2+^ is critically important in developing a rapid screening protocol. Genotypic differences in relative plant growth were evident at 100–150 μM Mn, after 14 days of Mn treatments. Among all of the Mn treatments, 150 μM Mn showed the best discrimination into tolerant and intolerant genotypes with respect to relative shoot and root growth measured after 14 days of Mn treatments. At 14 days, the domestic genotypes Ambar, PBA Striker, and *C. echinospermum* were more tolerant than PBA HatTrick and *C. reticulatum*. The relative plant growth (shoot and root) after 14 days in 150 μM Mn solution for genotypes Ambar (79%), PBA Striker (75%) and *C. echinospermum* (71%) were significantly higher than that for PBA HatTrick (59%) and *C. reticulatum* (53%).

In all *Cicer* genotypes, relative root dry weight was more sensitive than relative shoot weight at Mn levels ≥150 μM at both harvests. Previous studies suggested that shoot injury due to excess Mn indirectly affects root growth, but more pronounced root growth reduction than shoot growth was reported for other plant species like wheat (Khabaz-Saberi et al., 2010). In contrast, in soybean 300 μM Mn did not affect root growth but reduced shoot dry matter significantly (Santos et al., 2017) and similarly in triticale (Quartin et al., 1998) and perennial ryegrass (Mora et al., 2009). However, rapeseed showed equal reduction in both shoot and root growth due to Mn stress (Moroni et al., 2003). Even though in the present study, root growth was more severely affected than shoot growth in *Cicer* species, shoot dry weight data were more accurate and easier to sample than those for roots. Furthermore, if soils are used for Mn toxicity rankings among genotypes, shoot dry weight can be more easily used than root dry weight.

Generally, in *Cicer*, Mn^2+^ ions were more concentrated in roots than in shoots, similar to clover (*Trifolium repens* L.) (Rosas et al., 2007) and ryegrass (*Lolium perenne* L.) (Mora et al., 2009). By contrast, in rice, leaves had higher Mn concentration than roots (Lidon, 2001; Millaleo et al., 2010). In this study, tolerant genotypes PBA Striker and *C. echinospermum* accession had high shoot/root Mn concentrations, indicating the increase in translocation of Mn from roots to shoots to a greater extent than that in sensitive *C. reticulatum*, while the shoot/root Mn accumulation or uptake per plant was significantly higher in tolerant genotypes PBA Striker and *C. echinospermum* than in other *Cicer* genotypes at first harvest. The Mn^2+^ taken up by roots is translocated to various shoot tissues; therefore, identification of long-distance transport regulation in *Cicer* could greatly improve the understanding of Mn tolerance mechanisms.

Manganese toxicity in plants can prevent uptake and translocation of other essential elements like Ca, Mg, Fe, and P (Blamey et al., 2015; Alejandro et al., 2020). In this experiment, Mn toxicity depressed Ca^2+^ concentrations in shoots of *Cicer* sp., suggesting competition between these two cations for specific absorption and translocation. Indeed, most of the Mn^2+^ transporters are non-specific and are able to transport other divalent cations like Ca^2+^, Zn^2+^, and Fe^2+^ (Alejandro et al., 2020). A similar decrease in Ca concentrations due to Mn toxicity was seen in barley plants (Alam et al., 2001). Even though there was a decrease in Ca^2+^ concentrations due to excess Mn in the solutions, the Ca concentrations in *Cicer* were adequate (Reuter and Robinson, 1997).

Excess Mn can sometimes produce symptoms that resemble Fe deficiency. Moreover, the addition of Fe alleviated Mn toxicity in beans and sunflower (Foy et al., 1988; Blamey et al., 2019). Among soybean cultivars, there were contrasting response of plant Fe to excess Mn due to genotypic differences within soybean which influence the expression of Mn toxicity symptoms (Izaguirre-Mayoral and Sinclair, 2005). In the present experiment, wild *C. reticulatum* and *C. echinospermum* accessions concentrated more Fe in shoots even under Mn toxicity, even though they showed more intense Mn toxicity symptoms compared with the *Cicer* cultivars.

Mean Mn concentrations of *Cicer* genotypes in control solution were between 120 and 300 mg/kg depending on plant tissue analyzed, and in 200 μM Mn solution, Mn concentrations were 3,500, 5,000, and 6,300 mg/kg in young leaves, old leaves, and roots, respectively. Lupin had Mn concentrations of around 6,000 to 7,000 mg/kg in young shoots and roots; however, old leaves had >10,000 mg/kg at 200 μM Mn. At a similar solution concentration of 200 μM Mn, canola (*B. napus* L.) contained an Mn concentration of 3,500 mg/kg (Moroni et al., 2003), similar to that of chickpeas, while soybean contained only 886 mg/kg in shoots (Lavres Junior et al., 2010). By comparison, clover contained up to 2,050 mg/kg in shoots and 7,481 mg/kg Mn in roots when grown at 355 μM Mn (Rosas et al., 2007).

In the present study, better Mn toxicity tolerance in cv. PBA Striker was associated with greater uptake and transport of Mn from roots to shoots than were sensitive *C. reticulatum* accession and cv. PBA HatTrick. Also, PBA Striker had higher shoot Ca and Mn concentrations and uptake compared with *C. reticulatum*, although it had reduced Fe concentrations in young leaves compared to sensitive wild *C. reticulatum* accession. Among the wild accessions, *C. echinospermum*, which showed better Mn toxicity tolerance, had higher shoot Mn concentrations compared with the *C. reticulatum* accession. Therefore, in *Cicer* genotypes, tolerant genotypes accumulated more Mn in shoots, suggesting that internal sequestration in shoots and tolerance to excess Mn are the main mechanisms of tolerance. Manganese is known to complex with organic compounds in less active plant cells or organelles like vacuoles, endoplasmic reticulum (Lidon et al., 2004; Millaleo et al., 2010; Li et al., 2019), and cell walls or is complexed by organic acids in different leaf tissues (Alejandro et al., 2020). In this respect, *Cicer* is similar to Mn-tolerant wheat (Khabaz-Saberi et al., 2010), rapeseed (Moroni et al., 2003), and maize genotypes (Stoyanova et al., 2009).

In spite of chickpea being a significant pulse crop and acidity being a major limitation for expanding its production areas, there is limited research on Mn toxicity in chickpea. The protocol developed here can be used to screen a wide range of released cultivars of chickpea and wild *Cicer* germplasm accessions to identify Mn-tolerant genotypes. Confirmation of Mn toxicity tolerance in soils is needed, but screening in soils in the field has proved unreliable due to the range of soil factors that alter soil Mn^2+^ concentrations, the level of mycorrhizal association in roots, and root rhizosphere effects (Nogueira and Cardoso, 2003; Moroni et al., 2009). The mycorrhizal association may alleviate Mn toxicity if the association is effective (Nogueira and Cardoso, 2003; Nogueira et al., 2004). Further research on linking phenotypic Mn toxicity tolerance responses with genomic responses may produce molecular markers for screening programs for Mn toxicity tolerance in *Cicer*. As *Cicer* was found to accumulate and tolerate high Mn under stress, identifying the key transporter genes responsible would increase our understanding of how they acquire, transport, and tolerate excess Mn.

## Conclusion

Among the current prominent chickpea cultivars in Australia, PBA Striker was more tolerant than PBA HatTrick, suggesting that there is genotypic variation in Mn toxicity tolerance among chickpea cultivars currently grown in Australia. Moreover, neither of the accessions screened from *C. reticulatum* and *C. echinospermum* showed greater Mn toxicity tolerance than domestic cultivar PBA Striker. The limitations in both shoot and root dry matter in Mn-sensitive genotypes can be used as a selection criterion to screen genotypes for Mn toxicity, and the best discrimination into tolerant and intolerant *Cicer* genotypes was achieved at 100–200 μM Mn grown in Mn treatments for 14 days. Therefore, 150 μM Mn and 14 days of growth in low-ionic-strength nutrient solution at pH 5.2 are proposed for future large-scale screening of *Cicer* germplasm. The scoring of symptoms at 7 and 14 days was useful in domestic cultivars; however, in wild accessions, scoring did not distinguish among genotypes as well as differences in their dry weights. Chlorophyll fluorescence measurements were not a useful selection criterion for Mn toxicity differentiation among genotypes in this experiment. Manganese toxicity effects were not related to induced Ca or Fe deficiency even though increasing Mn had a detrimental effect on Ca concentrations and uptake. Manganese tolerance in *Cicer* genotypes was associated with greater uptake of Mn and translocation of Mn from roots to shoots, suggesting that their internal tolerance to excess Mn was the main mechanism of Mn toxicity tolerance.

## Data Availability Statement

The original contributions presented in the study are included in the article/Supplementary Material, further inquiries can be directed to the corresponding author/s.

## Author Contributions

KP designed and conducted this study under the supervision of RB and WV. KP wrote the original manuscript with revisions provided by RB and WV. All authors discussed the results and reviewed the manuscript.

## Conflict of Interest

The authors declare that the research was conducted in the absence of any commercial or financial relationships that could be construed as a potential conflict of interest.

## Footnotes

1 Australian Grain Genebank (AGG) accession number 49936 is a *C. reticulatum* from Turkey; collection site Egil with AGG account name Egil_063, from Diyarbakir Province and Egil District; latitude: 38.2745, longitude: 40.06454; elevation: 987.68 m asl.

2 AGG accession number 50166 is a *C. echinospermum* from Turkey; collection site Kara with AGG account name Karab_162, from Urfa Province and Siverek District; latitude: 37.82429, longitude: 39.76289; elevation: 1,266.71 m asl.
