# Supplementary material for: Variation of Cicer Germplasm to Manganese Toxicity Tolerance
Source: Front Plant Sci. 2020 Nov 30;11:588065. doi: 10.3389/fpls.2020.588065 (PMC7733998; doi:10.3389/fpls.2020.588065)
Supplement: Supplementary file 1 [file Data_Sheet_1.docx]

**Appendix A Supplementary file**

**Variation of *Cicer* germplasm to manganese toxicity tolerance**

**Table- A** Composition of nutrient solution used in this study in comparison to previous relevant Mn screening studies

| Mn studies | (Blamey et al., 2015) | (Moroni et al., 1999) | (Foy et al., 1995) | (Khabaz-Saberi et al., 2010) | (Rout et al., 2001) | (Stoyanova et al., 2009) | (Shrestha et al., 2018) | (Quartin et al., 1998) | This study |
| --- | --- | --- | --- | --- | --- | --- | --- | --- | --- |
| Plant species | Soybean, lupin and sunflower | Rapeseed | Cotton | Wheat | Mung bean and rice | Maize | Rice | Triticale | Chickpea |
| Macronutrients (μM) | | | | | | | | | |
| N as NO_3_ | 670 | 3400 | 3710 | 3300-3600 | 8000 | 700 | 1428 | 3000 | 1000 |
| N as NH_4_ | 120 | 600 | 300 | 600 | 800 | 300 | 1428 | 200 | 160 |
| total N | 790 | 4000 | 4010 | 3900-4200 | 8800 | 1000 | 2856 | 3200 | 1160 |
| P | 5 | 100 | 100 | 100 | 143 | 5 | 310 | 125 | 60 |
| K | 300 | 1000 | 750 | 800 | 4000 | 400 | 1022 | 1125 | 700 |
| Ca | 1000 | 1000 | 1270 | 1000 | 4000 | 200 | 998 | 1000 | 450 |
| Mg | 95 | 400 | 270 | 300 | 2000 | 100 | 1646 | 500 | 200 |
| S | 340 | 401 | 120 | 100 | 2400 | 306 | 2420 | 700 | 550 |
| Micronutrients (μM) | | | | | | | | | |
| Cl | 1250 | 78 | 58.5 | 34 | 30 | 0 | - | 60 | 60 |
| Cu | 0.2 | 0.3 | 0.2 | 0.15 | 0.3 | 0.16 | 0.16 | 0.3 | 0.2 |
| Zn | 0.5 | 0.8 | 0.6 | 0.5 | 0.8 | 0.38 | 0.15 | 0.8 | 0.6 |
| B | 1 | 23 | 6.6 | 6 | 10 | 8 | 18.5 | 10 | 23 |
| Mo | 0.01 | 0.1 | 0.1 | 0.01 | 0.4 | 0.42 | 0.52 | 0.1 | 0.1 |
| Fe | 6 | 20 | 17.9 | 10 | 20 | 10 | 35.8 | 10 | 20 |
| Na | 10 | 40.2 | 53.9 | 20 | 30 | 5 | - | 30 | 40 |
| Mn treatments (µM ) | 0.5, 30 &100 | 2, 50, 100, 150, 200, 300, 400, 500; 9 & 125 | 72, 145 & 291 | 2, 250, 500, 750, 1000, 2000 & 3000  Screening: 2 & 750 | 2.0, 71, 142, 284, 568 & 1136 | 5, 50, 200 and 500 | 9.1 and 91 | 36.4, 50, 200, 300, 400 & 600; 182, 910, 1820 & 3640 | 2, 25, 50, 100, 150, 200, 250 & 500 |
| pH | 5.6 | 4.7 | 4.6 & 5 | 4.8 | 6.8 | - | 5.2-5.5 | 4.5 | 5.2 |

**Table- B** Scoring of *Cicer* plants for Mn toxicity symptoms with brief description of symptoms

| Score | Symptoms | Description |
| --- | --- | --- |
| 0 | No symptoms | 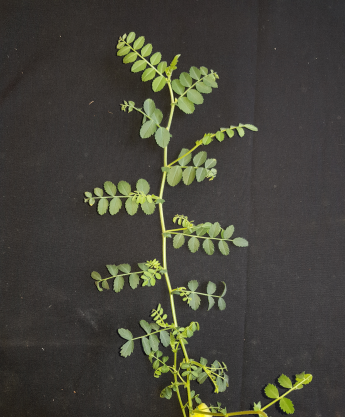  The plants were healthy. No deficiency or toxicity symptoms noticed. The leaves were healthy and green |
| 1 | Very mild | 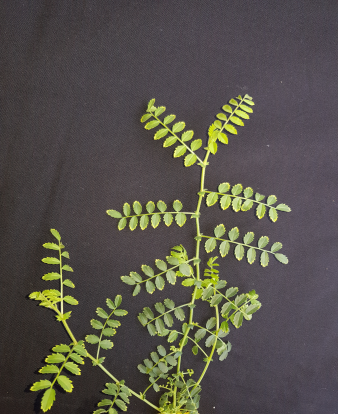  Young leaves were pale green in colour. Leaf tips and serrated margins close to the leaf tips turned slightly yellow.  Overall plants were healthy and showed very mild symptoms |
| 2 | Mild | 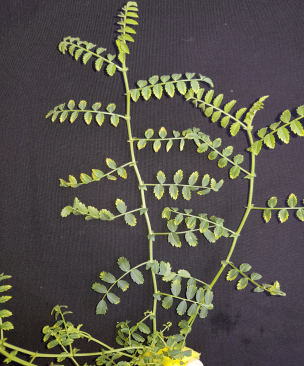  Leaf tips and serrated margins of the young leaves turned yellow, and showed Mn toxicity symptoms.  Young leaves were pale green in colour similar to Fe deficiency symptoms.  Overall the plants were healthy |
| 3 | Moderate | 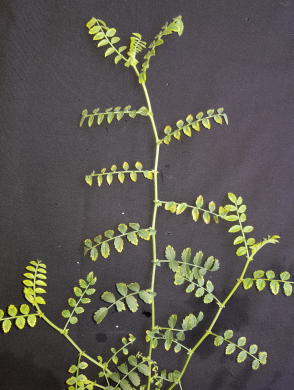  Young leaves turned pale. Leaf tips and margins turned yellow. Brown necrotic areas developed along the leaf margins and leaf size were reduced compared to healthy plants |
| 4 | Severe | 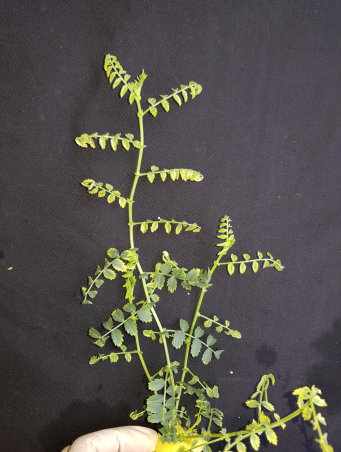Young leaves were severely affected. Brown necrotic areas enlarged throughout the leaf and the leaf size was considerably reduced. The leaves were shrivelled, curled and fell from the leaf if disturbed |
| 5 | Very severe | 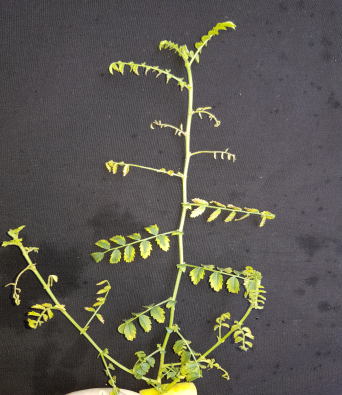  The whole plant was very severely affected and growth was significantly reduced. Brown necrotic areas enlarged throughout the leaf and the leaf size was very much reduced. The leaves dropped from the plant readily |

**Table C** Ca, Fe and Mn uptake per plant (mg/plant) in *Cicer* genotypes and lupin in shoots and roots at both harvests, 14 and 28 days after Mn treatments

| Ion content (mg/plant) of *Cicer* and lupin in shoot at 14 days after Mn treatment | | | | | | | | | | | | | | | | | | | | | | |
| --- | --- | --- | --- | --- | --- | --- | --- | --- | --- | --- | --- | --- | --- | --- | --- | --- | --- | --- | --- | --- | --- | --- |
|  | **2 Mn** | | | | **100 Mn** | | | | | | **150 Mn** | | | | | | **200 Mn** | | | | | |
| Cultivar | *Ca* | *Fe* | *Mn* | | *Ca* | | *Fe* | | *Mn* | | *Ca* | | *Fe* | | *Mn* | | *Ca* | | *Fe* | | *Mn* | |
| Ambar | 10.0 | 0.14 | 0.17 | | 6.94 | | 0.09 | | 2.05 | | 5.13 | | 0.06 | | 2.07 | | 3.69 | | 0.04 | | 2.09 | |
| HatTrick | 8.34 | 0.13 | 0.12 | | 4.60 | | 0.07 | | 1.38 | | 3.50 | | 0.06 | | 1.31 | | 3.21 | | 0.05 | | 1.88 | |
| Striker | 11.3 | 0.12 | 0.15 | | 7.15 | | 0.08 | | 2.09 | | 5.13 | | 0.08 | | 2.15 | | 4.76 | | 0.06 | | 2.70 | |
| *C.retic* | 6.58 | 0.09 | 0.07 | | 2.96 | | 0.06 | | 0.75 | | 2.39 | | 0.06 | | 0.86 | | 1.83 | | 0.04 | | 0.91 | |
| *C.echino* | 8.07 | 0.11 | 0.08 | | 4.34 | | 0.08 | | 1.24 | | 4.29 | | 0.09 | | 1.77 | | 3.33 | | 0.05 | | 1.78 | |
| Lupin | 7.75 | 0.14 | 0.17 | | 5.45 | | 0.01 | | 1.57 | | 4.66 | | 0.08 | | 1.42 | | 6.56 | | 0.06 | | 3.72 | |
| Ion content (mg/plant) *Cicer* and lupin in shoot at 28 days after Mn treatment | | | | | | | | | | | | | | | | | | | | | | |
|  | **2 Mn** | | | | **100 Mn** | | | | | | **150 Mn** | | | | | | **200 Mn** | | | | | |
| Cultivar | *Ca* | *Fe* | *Mn* | | *Ca* | | *Fe* | | *Mn* | | *Ca* | | *Fe* | | *Mn* | | *Ca* | | *Fe* | | *Mn* | |
| Ambar | 21.2 | 0.26 | 0.43 | | 10.3 | | 0.14 | | 3.23 | | 10.6 | | 0.12 | | 4.62 | | 6.97 | | 0.10 | | 4.24 | |
| HatTrick | 15.8 | 0.20 | 0.31 | | 7.58 | | 0.13 | | 2.37 | | 6.49 | | 0.10 | | 2.83 | | 4.85 | | 0.08 | | 3.08 | |
| Striker | 18.2 | 0.19 | 0.26 | | 8.28 | | 0.14 | | 2.48 | | 8.27 | | 0.13 | | 3.86 | | 6.50 | | 0.13 | | 4.06 | |
| *C.retic* | 10.7 | 0.14 | 0.16 | | 4.01 | | 0.09 | | 1.18 | | 2.75 | | 0.06 | | 1.13 | | 2.65 | | 0.07 | | 1.51 | |
| *C.echino* | 16.4 | 0.18 | 0.22 | | 9.06 | | 0.23 | | 2.79 | | 6.18 | | 0.16 | | 2.79 | | 6.39 | | 0.14 | | 3.93 | |
| Lupin | 18.0 | 0.21 | 0.43 | | 9.79 | | 0.18 | | 3.30 | | 9.83 | | 0.12 | | 4.57 | | 9.14 | | 0.17 | | 5.59 | |
| Ion content (mg/plant) of *Cicer* and lupin in root at 14 days after Mn treatment | | | | | | | | | | | | | | | | | | | | | |  |
|  | **2 Mn** | | | **100 Mn** | | | | | | **150 Mn** | | | | | | **200 Mn** | | | | | |  |
| Cultivar | *Ca* | *Fe* | *Mn* | *Ca* | | *Fe* | | *Mn* | | *Ca* | | *Fe* | | *Mn* | | *Ca* | | *Fe* | | *Mn* | |  |
| Ambar | 1.89 | 1.02 | 0.25 | 1.51 | | 1.11 | | 1.50 | | 1.60 | | 1.26 | | 1.66 | | 1.16 | | 1.26 | | 1.72 | |  |
| HatTrick | 1.70 | 0.80 | 0.16 | 1.21 | | 0.76 | | 1.08 | | 0.95 | | 0.69 | | 0.79 | | 0.93 | | 0.91 | | 1.63 | |  |
| Striker | 2.23 | 0.91 | 0.08 | 1.45 | | 0.85 | | 1.32 | | 1.33 | | 0.89 | | 1.24 | | 1.35 | | 0.79 | | 2.12 | |  |
| *C.retic* | 1.28 | 0.91 | 0.08 | 0.78 | | 0.98 | | 0.87 | | 0.47 | | 0.50 | | 0.55 | | 0.40 | | 0.46 | | 0.84 | |  |
| *C.echino* | 1.67 | 0.75 | 0.05 | 1.15 | | 0.75 | | 0.58 | | 1.01 | | 0.66 | | 0.74 | | 0.86 | | 0.61 | | 1.06 | |  |
| Lupin | 1.14 | 0.30 | 0.10 | 0.74 | | 0.31 | | 0.57 | | 0.69 | | 0.33 | | 0.62 | | 0.95 | | 0.59 | | 1.30 | |  |
| Ion content (mg/plant) of *Cicer* and lupin in root at 28 days after Mn treatment | | | | | | | | | | | | | | | | | | | | | |  |
|  | **2 Mn** | | | **100 Mn** | | | | | | **150 Mn** | | | | | | **200 Mn** | | | | | |  |
| Cultivar | *Ca* | *Fe* | *Mn* | *Ca* | | *Fe* | | *Mn* | | *Ca* | | *Fe* | | *Mn* | | *Ca* | | *Fe* | | *Mn* | |  |
| Ambar | 3.86 | 1.98 | 0.39 | 2.45 | | 2.71 | | 1.06 | | 2.08 | | 2.02 | | 1.73 | | 1.41 | | 1.62 | | 1.35 | |  |
| HatTrick | 2.87 | 1.43 | 0.21 | 2.20 | | 1.90 | | 1.04 | | 1.65 | | 1.23 | | 1.19 | | 0.98 | | 0.83 | | 0.99 | |  |
| Striker | 3.90 | 1.82 | 0.22 | 2.60 | | 2.12 | | 1.16 | | 2.04 | | 1.62 | | 1.02 | | 1.51 | | 1.38 | | 1.44 | |  |
| *C.retic* | 2.44 | 1.34 | 0.20 | 1.38 | | 1.39 | | 0.81 | | 0.90 | | 0.84 | | 0.79 | | 0.61 | | 0.61 | | 0.98 | |  |
| *C.echino* | 3.86 | 1.57 | 0.16 | 2.61 | | 2.21 | | 1.01 | | 1.65 | | 1.52 | | 1.21 | | 1.45 | | 1.23 | | 1.60 | |  |
| Lupin | 1.65 | 0.65 | 0.10 | 1.13 | | 0.45 | | 0.77 | | 0.60 | | 0.20 | | 0.51 | | 0.90 | | 0.31 | | 1.43 | |  |

*Mn/Ca ratios*

Mn/ Ca ratios in shoot and root increased with increase in Mn levels in treatment solutions. The mean Mn/Ca ratio in shoots at first harvest with Mn treatments 100 to 200 Mn, of all five *Cicer* genotypes was around 0.4. At second harvest, there was slight consistent increase in shoot Mn/Ca ratios of *Cicer* genotypes. The mean shoot Mn/Ca ratios for Wild *C.retic* accession with Mn treatments 100 to 200 μM Mn was significantly low than domestic *Cicer* cultivars, however, the mean root Mn/Ca ratios was higher in *C.retic* than domestic cultivars and *C.echino* accession at both harvests (*P* ≤ 0.05).

**Table D** Mn/Ca ratios in *Cicer* genotypes and lupin in shoots and roots grown in solution at 2, 100, 150 and 200 μM Mn for 14 and 28 days after Mn treatments

| Mn/Ca ratios at first harvest/ 14 days after Mn treatment | | | | | | | | | | |
| --- | --- | --- | --- | --- | --- | --- | --- | --- | --- | --- |
|  | Shoot Mn/Ca | | | | | Root Mn/Ca | | | | |
| Mn levels μM | 2 | 100 | 150 | | 200 | 2 | 100 | 150 | | 200 |
| Ambar | 0.01 | 0.30 | 0.40 | | 0.57 | 0.13 | 1.01 | 1.04 | | 1.55 |
| HatTrick | 0.01 | 0.30 | 0.37 | | 0.60 | 0.09 | 0.91 | 0.80 | | 1.75 |
| Striker | 0.01 | 0.29 | 0.41 | | 0.56 | 0.04 | 0.91 | 0.92 | | 1.57 |
| *C.retic* | 0.01 | 0.25 | 0.36 | | 0.50 | 0.06 | 1.12 | 1.11 | | 2.03 |
| *C.echino* | 0.01 | 0.28 | 0.41 | | 0.53 | 0.03 | 0.50 | 0.71 | | 1.23 |
| Lupin | 0.02 | 0.29 | 0.31 | | 0.56 | 0.08 | 0.79 | 0.89 | | 1.40 |
|  | *P* values | | | LSD_0.05_ values | | *P* values | | | LSD_0.05_ values | |
| Mn treatment | 0.000 | | | 0.04 | | 0.002 | | | 0.37 | |
| Genotypes | 0.08 | | | - | | 0.01 | | | 0.22 | |
| Mn*genotypes | 0.32 | | | - | | 0.82 | | | - | |
| Mn/Ca ratios at second harvest/ 28 days after Mn treatment | | | | | | | | | | |
|  | Shoot Mn/Ca | | | | | Root Mn/Ca | | | | |
| Mn levels μM | 2 | 100 | 150 | | 200 | 2 | 100 | 150 | | 200 |
| Ambar | 0.02 | 0.31 | 0.43 | | 0.61 | 0.11 | 0.43 | 0.85 | | 0.99 |
| HatTrick | 0.02 | 0.32 | 0.43 | | 0.66 | 0.07 | 0.49 | 0.74 | | 0.92 |
| Striker | 0.01 | 0.30 | 0.46 | | 0.63 | 0.05 | 0.47 | 0.47 | | 0.98 |
| *C.retic* | 0.01 | 0.29 | 0.41 | | 0.57 | 0.08 | 0.64 | 1.00 | | 1.66 |
| *C.echino* | 0.01 | 0.31 | 0.43 | | 0.62 | 0.04 | 0.39 | 0.72 | | 1.07 |
| Lupin | 0.02 | 0.34 | 0.45 | | 0.58 | 0.05 | 0.70 | 0.91 | | 1.37 |
|  | *P* values | | | LSD_0.05_ values | | *P* values | | | LSD_0.05_ values | |
| Mn treatment | 0.000 | | | 0.06 | | 0.002 | | | 0.37 | |
| Genotypes | 0.04 | | | 0.02 | | 0.02 | | | 0.22 | |
| Mn*genotypes | 0.17 | | | - | | 0.64 | | | - | |

*Mn/Fe ratios*

There was significant interaction between Mn treatments and cultivars (*P* ≤ 0.05) for shoot Mn/Fe ratios at first harvest (Table 5). In general, wild accessions had lower Mn/Fe ratios especially in shoots compared to domestic cultivars, *C.retic* accession had low shoot Mn/Fe compared to other genotypes, followed by *C.echino*. However, root Mn/Fe at first harvest did not show significant cultivar effects, except *C.retic* had lower root Mn/Fe ratios than Ambar. Similar to first harvest, shoot Mn/Fe ratios at 28 days after Mn treatments showed significant interaction among the cultivars; the genotypes *C.retic* and *C.echino* had significantly low shoot mean Mn/Fe ratios at second harvest than domestic cultivars. The mean root Mn/Fe ratios at second harvest was affected by Mn treatments, however, there was no significant difference in root Mn/Fe ratios among the *Cicer* genotypes.

**Table E** Mn/Fe ratios in *Cicer* genotypes and lupin grown in solution at 2, 100, 150 and 200 μM Mn for 14 and 28 days after Mn treatments

| Mn/Fe ratios at 14 days after Mn treatment | | | | | | | | | | | |
| --- | --- | --- | --- | --- | --- | --- | --- | --- | --- | --- | --- |
|  | Shoot Mn/Fe | | | | | Root Mn/Fe | | | | | |
| Mn levels μM | 2 | 100 | | 150 | 200 | 2 | 100 | | 150 | | 200 |
| Ambar | 1.03 | 23.8 | | 33.8 | 44.5 | 0.32 | 1.41 | | 1.62 | | 2.59 |
| HatTrick | 0.95 | 21.1 | | 22.0 | 36.0 | 0.22 | 1.52 | | 1.25 | | 2.86 |
| Striker | 1.19 | 26.0 | | 27.4 | 44.8 | 0.09 | 1.77 | | 1.40 | | 2.69 |
| *C.retic* | 0.72 | 13.2 | | 14.6 | 23.6 | 0.09 | 1.32 | | 1.31 | | 2.32 |
| *C.echino* | 0.76 | 16.6 | | 18.6 | 40.3 | 0.07 | 0.78 | | 1.14 | | 2.36 |
| Lupin | 1.22 | 19.4 | | 17.4 | 69.1 | 0.40 | 1.94 | | 2.13 | | 2.25 |
|  | *P* values | | LSD_0.05_ | | | *P* values | | LSD_0.05_ | | | |
| Mn treatment | 0.000 | | - | | | 0.08 | | - | | | |
| Genotypes | 0.000 | | - | | | 0.35 | | - | | | |
| Mn*genotypes | 0.001 | | within Mn:12.4, between Mn: 15.1 | | | 0.95 | | - | | | |
| Mn/Fe ratios at 28 days after Mn treatment | | | | | | | | | | | |
|  | Shoot Mn/Fe | | | | | Root Mn/Fe | | | | | |
| Mn levels μM | 2 | 100 | | 150 | 200 | 2 | 100 | | 150 | | 200 |
| Ambar | 1.73 | 24.3 | | 36.8 | 42.0 | 0.22 | 0.42 | | 0.85 | | 0.84 |
| HatTrick | 1.74 | 18.5 | | 26.0 | 34.5 | 0.14 | 0.64 | | 0.97 | | 1.14 |
| Striker | 1.36 | 18.2 | | 29.8 | 31.5 | 0.17 | 0.81 | | 0.62 | | 1.24 |
| *C.retic* | 1.22 | 13.2 | | 18.4 | 23.0 | 0.15 | 0.69 | | 1.01 | | 1.57 |
| *C.echino* | 1.34 | 12.7 | | 17.4 | 29.5 | 0.12 | 0.46 | | 0.78 | | 1.25 |
| Lupin | 1.98 | 21.9 | | 38.2 | 44.5 | 0.14 | 1.63 | | 2.56 | | 3.59 |
|  | *P* values | | LSD_0.05_ | | | *P* values | | | | LSD_0.05_ | |
| Mn treatment | 0.000 | | - | | | 0.000 | | | | 0.14 | |
| Genotypes | 0.000 | | - | | | 0.000 | | | | 0.49 | |
| Mn*genotypes | 0.02 | | within Mn: 8, between Mn: 11 | | | 0.109 | | | | - | |
